# Intense and Mild First Epidemic Wave of Coronavirus Disease, The Gambia

**DOI:** 10.3201/eid2708.204954

**Published:** 2021-08

**Authors:** Baderinwa Abatan, Orighomisan Agboghoroma, Fatai Akemoke, Martin Antonio, Babatunde Awokola, Mustapha Bittaye, Abdoulie Bojang, Kalifa Bojang, Helen Brotherton, Carla Cerami, Ed Clarke, Umberto D’Alessandro, Thushan de Silva, Mariama Drammeh, Karen Forrest, Natalie Hofmann, Sherifo Jagne, Hawanatu Jah, Sheikh Jarju, Assan Jaye, Modou Jobe, Beate Kampmann, Buba Manjang, Melisa Martinez-Alvarez, Nuredin Mohammed, Behzad Nadjm, Mamadou Ousmane Ndiath, Esin Nkereuwem, Davis Nwakanma, Francis Oko, Emmanuel Okoh, Uduak Okomo, Yekini Olatunji, Eniyou Oriero, Andrew M. Prentice, Charles Roberts, Anna Roca, Babanding Sabally, Sana Sambou, Ahmadou Samateh, Ousman Secka, Abdul Karim Sesay, Yankuba Singhateh, Bubacarr Susso, Effua Usuf, Aminata Vilane, Oghenebrume Wariri

**Affiliations:** Medical Research Council Unit The Gambia at the London School of Hygiene and Tropical Medicine, London, UK (B. Abatan, O. Agboghoroma, F. Akemoke, M. Antonio, B. Awokola, A. Bojang, K. Bojang, H. Brotherton, C. Cerami, E. Clarke, U. D’Alessandro, T. de Silva, K. Forrest, N. Hofmann, H. Jah, S. Jarju, A. Jaye, M. Jobe, B. Kampmann, M. Martinez-Alvarez, N. Mohammed, B. Nadjm, M.O. Ndiath, E. Nkereuwem, D. Nwakanma, F. Oko, E. Okoh, U. Okomo, Y. Olatunji, E. Oriero, A.M. Prentice, A. Roca, O. Secka, A.K. Sesay, B. Susso, E. Usuf, A. Vilane, O. Wariri);; Ministry of Health, Banjul, The Gambia (M. Bittaye, M. Drammeh, S. Jagne, B. Manjang, C. Roberts, B. Sabally, A. Samateh, S. Sambou, Y. Singhateh)

**Keywords:** Africa, The Gambia, transmission rate, disease burden, severity respiratory infections, severe acute respiratory syndrome coronavirus 2, SARS-CoV-2, SARS, COVID-19, coronavirus disease, zoonoses, viruses, coronavirus

## Abstract

The severe acute respiratory syndrome coronavirus 2 (SARS-CoV-2) pandemic is evolving differently in Africa than in other regions. Africa has lower SARS-CoV-2 transmission rates and milder clinical manifestations. Detailed SARS-CoV-2 epidemiologic data are needed in Africa. We used publicly available data to calculate SARS-CoV-2 infections per 1,000 persons in The Gambia. We evaluated transmission rates among 1,366 employees of the Medical Research Council Unit The Gambia (MRCG), where systematic surveillance of symptomatic cases and contact tracing were implemented. By September 30, 2020, The Gambia had identified 3,579 SARS-CoV-2 cases, including 115 deaths; 67% of cases were identified in August. Among infections, MRCG staff accounted for 191 cases; all were asymptomatic or mild. The cumulative incidence rate among nonclinical MRCG staff was 124 infections/1,000 persons, which is >80-fold higher than estimates of diagnosed cases among the population. Systematic surveillance and seroepidemiologic surveys are needed to clarify the extent of SARS-CoV-2 transmission in Africa.

By the end of October 2020, the severe acute respiratory syndrome coronavirus 2 (SARS-CoV-2) pandemic had spread to 6 continents and caused >45 million coronavirus disease (COVID-19) cases and 1.1 million deaths (*1*). Despite having 15.6% of the worldwide population (*2*), by October 31, 2020, Africa had only 3.9% (1.76 million) of the world’s COVID-19 cases and 3.6% (42,233) of deaths during the pandemic (*1*). Data suggest that the pandemic is evolving differently in sub-Saharan Africa compared with the rest of the world and that the outbreak started later (*3*).

Of note, severe COVID-19 cases seem to occur less frequently in Africa than in the rest of the world (*4*). Several factors have been proposed to explain this. Age is likely a major factor because older persons are at higher risk for severe disease, but Africa has an extremely young population; >60% of persons are <25 years of age (*5*). However, variation of COVID-19 severity with age alone does not fully explain the observed differences (*4*). Clinical cases and deaths in Africa likely are underreported because systematic surveillance is limited and no systematic death registration exists; thus, the true SARS-CoV-2 burden probably is underestimated (*4*). Nevertheless, local health systems in Africa, which have a lower capacity to deal with COVID-19 patients than healthcare systems in high-resource settings, were not overwhelmed, even at the peak of the epidemic (*6*). Although potential avoidance of medical care during the pandemic, as described in other regions (*7*), could partly explain the low number of hospitalized patients, the milder COVID-19 disease severity reported appears to be genuine, and several biologic and environmental factors have been proposed as potential contributing factors (*8*–*10*).

Recent serosurveys conducted in Kenya, Malawi, and South Africa showed that community transmission was several times higher than that detected by surveillance; 5%–40% of the population had SARS-CoV-2 IgG (*11*–*13*). Such results highlight the need for robust epidemiologic studies to assess the extent of community transmission in different regions in Africa.

The Gambia is the smallest country in continental mainland Africa and is surrounded by Senegal, except for its narrow Atlantic coast. Although an imported case was identified in The Gambia on March 17, 2020, by June 30, 2020, only 48 additional cases had been detected. Nevertheless, a rapid increase in cases was seen in July 2020, and by the end of September 2020, 3,579 cases were reported (*1*). The trajectory of the epidemic in The Gambia is different from that in Senegal, which has a population ≈7 times larger than The Gambia. In Senegal, community transmission was reported in early April 2020, and almost 7,000 cases were recorded by the end of June (*1*). Systematic surveillance, testing, contact tracing for staff of the Medical Research Council Unit The Gambia (MRCG) at the London School of Hygiene and Tropical Medicine (https://www.mrc.gm) who had influenza-like symptoms was implemented during the pandemic; the first case among MCRG staff was identified on July 18. We considered MRCG staff as a cohort to provide additional insights into the nature of the COVID-19 epidemic in The Gambia.

## Methods

### Population Demographics, Climate, and Healthcare Structure

**Figure 1:**
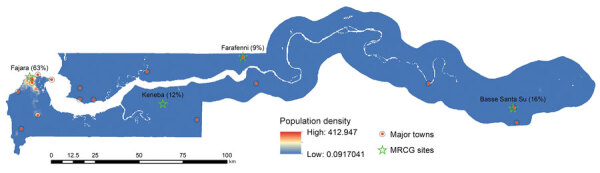
Population density of The Gambia, including Medical Research Council Unit The Gambia (MRCG) research sites distributed across the country.

In 2020, The Gambia had a population of ≈2.42 million. The median age is 17.8 years, and ≈41.9% of the population are 20–64 years of age. About 95% of the population is Muslim. The illiteracy rate is high across the country. Around 59% of the population live in urban and peri-urban settings, mainly along the coast (Figure 1). 

The climate is typical of the sub-Sahel region, including a long dry season during November–May and a short rainy season during June–October. Maximum temperature is high throughout the year, 30°C–34°C, and lowest during the rainy season; minimum temperatures range from 22°C–24°C during the rainy season to 16°C−20°C during the dry season (*14*). Humidity can be >80% during the rainy months (*15*).

The government of The Gambia is the main health provider, and healthcare delivery has 3 tiers, based on the primary healthcare strategy in which most healthcare delivery occurs at local health posts. The Gambia has 4 tertiary hospitals, 38 health centers at the secondary level, and 492 health posts at the primary level. The system is complemented by 34 private and nongovernmental organization clinics.

### COVID-19 Response in The Gambia

Shortly after the first COVID-19 case was detected in The Gambia on March 19, 2020, the country closed its international land, sea, and air borders. On March 27, the country declared a state of emergency, which included closing schools, nonessential shops, places of worship, and many workplaces. Initial SARS-CoV-2 testing by PCR was focused on identifying imported cases and tracing and isolating case contacts, especially among travelers from Senegal. The Ministry of Health, supported by several international organizations, set up a hotline for the public, which persons, including those with suspected cases, could call to ask for advice or request the surveillance team to perform the SARS-CoV-2 test either at health facilities or at home. 

As the epidemic progressed, the Ministry of Health established testing facilities at strategic locations in the most densely populated parts of the country, mainly the western urban areas. Persons were encouraged to go for testing if they were symptomatic or after contact with a confirmed COVID-19 case. Demand for testing services was not high, and attempts to raise awareness were unsuccessful. All identified cases were isolated in designated facilities regardless of symptoms until considered noninfectious as per World Health Organization (WHO) guidelines (*16*). Ministry of Health staff traced and quarantined contacts for 10 days in hotels during the early part of the outbreak, April–July 2020, after which persons were permitted to self-isolate for 10 days at home.

### MRCG Unit 

MRCG is a biomedical research institution that also provides outpatient and inpatient clinical care to the local population through its clinical services department (CSD). As of August 2020, MRCG had 1,336 employees. Staff were distributed as follows: 845 were along the coast, mainly in Fajara; 158 were in Keneba; 116 were in the Central River Division, mainly in Farafenni; and 217 were in the Upper River Division, mainly Basse (Figure 1). MRCG staff work in different environments, including 715 (53.5%) field-based staff, such as drivers, community workers, nurses, and research clinicians; 334 (25.0%) office-based staff, including those in administrative, operations, data-management, and statistics positions; and 177 (13.2%) laboratory-based staff. Only 110 (8.2%) MRCG staff provide healthcare to the general population at the CSD.

CSD is 1 of 2 hospital facilities in The Gambia able to care for severe COVID-19 patients. CSD dedicated 42 beds for COVID-19 patients, including MRCG staff and the general population. From the start of the epidemic, all staff were trained to wear appropriate personal protective equipment (PPE) according to international guidelines (*17*).

MRCG staff underwent a clinician-administered risk assessment in the early phases of the epidemic. Staff deemed to be at high risk for severe disease were advised to work from home and were excluded from high-risk clinical areas.

### Surveillance and Contact Tracing among MRCG Staff

In July 2020, MRCG established enhanced passive case detection by testing all staff exhibiting COVID-19 symptoms, such as cough, fever, headache, sore throat, nasal congestion, body pain, or other influenza-like symptoms. Families and contacts of symptomatic staff also were tested, as were staff known to have been exposed to confirmed cases. In addition, CSD staff were offered active weekly PCR-based testing, regardless of symptoms. MRCG set up a hotline manned by doctors from whom staff could receive answers to questions or concerns and get information on how to access services. Case contacts were called to confirm exposure and then tested 3–5 days after the last exposure. Regardless of negative test results, all exposed staff were quarantined for 14 days; SARS-CoV-2–positive staff isolated in their homes for 14 days, or at the MRCG site if at-home isolation was not possible, in line with WHO recommendations (*18*). 

### Sample Collection

Samples were collected via nasopharyngeal swab, oropharyngeal swab, or both by using FLOQSwabs (COPAN Diagnostics, https://www.copanusa.com). Samples were placed in single tubes containing universal transport medium (COPAN Diagnostics) and delivered to the laboratory within 24 hours. Sampling methods were comparable across cohorts with similar operational procedures and training.

### Laboratory Methods for SARS-CoV-2 Detection

MRCG laboratories collaborated with national public health laboratories to support national testing throughout the country during the epidemic. MRCG and these laboratories used the same laboratory methods and assays. Because the outbreak was expected to spread to the West Africa subregion, MRCG staff attended an Africa Centres for Disease Control and Prevention (https://africacdc.org) regional training workshop on diagnosing COVID-19, which was held in February 2020 in Dakar, Senegal. Thereafter, The Gambia established laboratory protocols for processing and testing suspected SARS-CoV-2–infected samples according to WHO guidelines (*19*,*20*). The same procedures and assays were transferred to the laboratory.

The standard test for COVID-19 diagnosis in The Gambia is real-time reverse transcription PCR (RT-PCR) of SARS-CoV-2–specific viral gene sequences. In the early stages of the outbreak, RT-PCR diagnosis was made by using the Berlin Charité Laboratory protocol (*21*), which targets the RNA-dependent RNA polymerase and envelope protein gene. Subsequent tests kits, primarily the Da An Gene Nucleic Acid Extraction Kit (Da An Gene Co., Ltd., of Sun Yat-sen University, https://en.daangene.com) and Novel Coronavirus (2019-nCoV) Nucleic Acid Diagnostic Kit (Sansure Biotech, Inc., http://eng.sansure.com.cn) were donated to the national public health libraries; both tests target the open reading frame 1ab and the nucleocapsid gene coding regions.

Sample inactivation and downstream RNA extraction were done by using commercially available kits according to the manufacturers’ protocols. Initial extractions were performed manually by using the QIAamp Viral RNA Mini Kit (QIAGEN, https://www.qiagen.com) or the IndiSpin Pathogen Kit (INDICAL BIOSCIENCE, https://www.indical.com). When donations to the public health system became available, kits from the Da An Gene Co., Ltd., of Sun Yat-sen University and Sansure Biotech, Inc., were included. As the outbreak progressed and daily sample numbers increased, automated RNA extraction system on the QIAcube HT (QIAGEN) was implemented. In all cases, 200 µL of universal transport medium sample was processed, and the RNA eluted in 50–80 µL, depending on the extraction kit. RT-PCR analysis was conducted with 5 µL of extracted RNA in 25 µL of reaction mix containing reaction buffer, one-step reverse transcription enzyme, either the Takara One Step PrimeScript III RT-PCR Kit (TaKaRa Bio, Inc., http://www.takara-bio.com) or SuperScript III Platinum One-Step qRT-PCR Kit (Invitrogen, https://www.thermofisher.com), and the primer and probe mix. 

Samples were defined as positive if amplification of any viral gene occurred after 40 cycles and with all the controls amplifying as appropriate. We defined a COVID-19 case as any person with a SARS-CoV-2–positive RT-PCR from an nasopharyngeal or oropharyngeal swab sample, regardless of symptomatology.

### Statistical Analysis

We calculated rates of risk for COVID-19 per 1,000 persons among the population of The Gambia. For MRCG, we stratified rates by occupational clinical exposure for staff working at the CSD versus non-CSD staff. In addition to occupational clinical exposure, surveillance for CSD staff was more intense due to routine testing, regardless of symptoms or known exposure.

The Ministry of Health generated daily national data for The Gambia (*22*). We extracted compiled data from the publicly available Johns Hopkins University COVID-19 database (*23*). The Gambian Government/MRCG Joint Ethics committee approved the study (reference no. L2020.E37).

## Results

Persons <25 years of age and persons >60 years of age are underrepresented in the MRCG cohort compared with the population of The Gambia (Table). In addition, urban residents are overrepresented in the MRCG cohort; 67.6% of MRCG staff live in cities or towns compared with 59.4% of the overall population.

**Table:** Epidemiologic and demographic characteristics of the population of The Gambia and staff of MRCG*

Baseline characteristics	The Gambia, no. (%)	MRCG staff, no. (%)
Age groups, y†		
<25	1,549,084 (64.2)	51 (3.89)
25–34	367,334 (15.2)	450 (34.35)
35–44	217,500 (9.0)	381 (29.08)
45–54	132,917 (5.5)	307 (23.44)
55–64	72,500 (3.0)	113 (8.63)
>65	74,917 (3.1)	8 (0.61)
Median age, y	17.8	37.5
Sex		
M	1,193,834 (49.4)	915 (68.5)
F	1,220,418 (50.6)	421 (31.5)
Living in main towns or cities‡	1,420,600 (59.4)	903 (67.6)

*MRCG, Medical Research Council Unit The Gambia at the London School of Hygiene and Tropical Medicine. †Ages were missing for 6 MRCG staff. ‡For MRCG staff location, we considered the workplace rather than the living place.

### SARS-CoV-2 Positivity Rates

**Figure 2:**
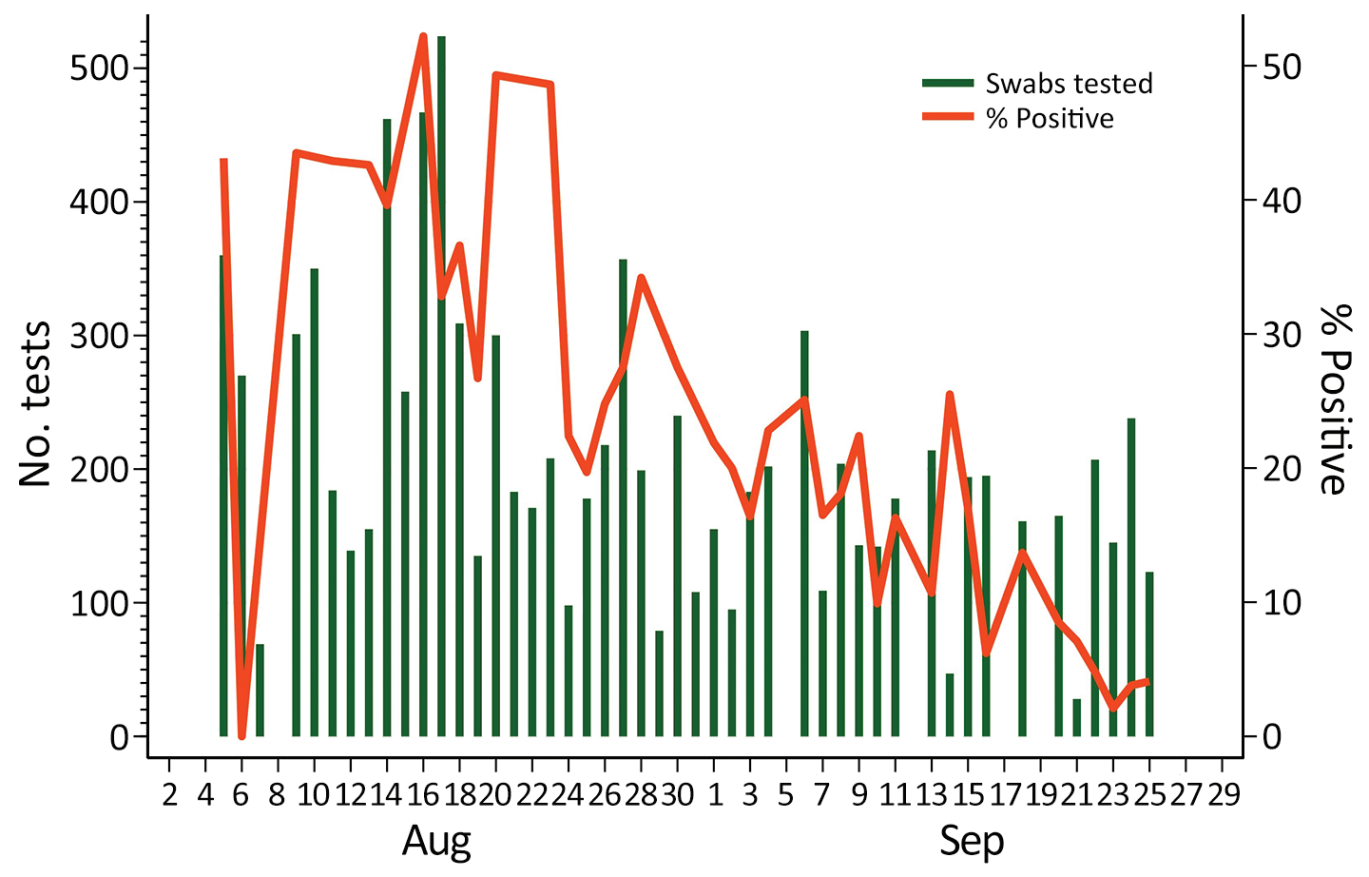
Number of daily nasopharyngeal and oropharyngeal swab samples tested for severe acute respiratory syndrome coronavirus 2 and percentage of positive samples in The Gambia during August–September 2020, the timeframe for the most intense transmission in the country.

From the start of the epidemic through September 30, 2020, a total of 17,885 samples were tested in The Gambia; 20.1% (3,590) were SARS-CoV-2–positive. The positivity rate was lower before July (1.6%; 40/3,095 samples tested) and higher during July–September (23.7%; 3,499/14,790 samples tested) (*19*,*20*). The number of samples collected and the positivity rate were the highest during August–September 2020, during which time the number of daily swabs collected varied from 28 to 524/day (median 184/day) (Figure 2). Positivity rate also varied substantially, from <5% to >50%. Approximately 67% of confirmed cases were detected in August; overall, 60% of confirmed cases were among persons <40 years of age (*20*).

During July 1–September 30, a total of 937 samples were collected from the MRCG cohort; 191 (20.4%) were SARS-CoV-2–positive. Most (60%) confirmed cases were detected in August. The median age among MRCG staff with SARS-CoV-2–positive samples was 36 years.

### Rates of Infection and Death

**Figure 3:**
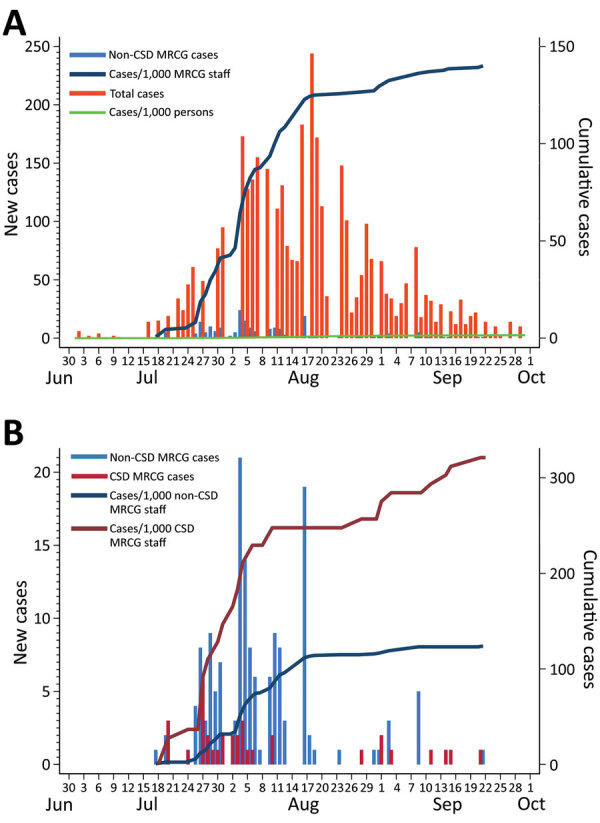
Daily COVID-19 cases and cumulative rates of SARS-CoV-2 infection per 1,000 persons among staff of Medical Research Council Unit The Gambia (MRCG) and the population of The Gambia, June 30–October 1, 2020. A) Case rates for MRCG staff outside the clinical service department and the population of The Gambia. We considered MRCG staff outside the clinical service department to be at the same risk for COVID-19 as the rest of the population. B) Risk for SARS-CoV-2 infection among MRCG staff stratified by potential occupational exposure risk. We considered clinical service department staff at highest risk for SARS-CoV-2 infection, and these staff were under more intense surveillance. Scales for the y-axes differ substantially to underscore patterns but do not permit direct comparisons. COVID-19, coronavirus disease; SARS-CoV-2, severe acute respiratory syndrome coronavirus 2.

By the end of September 2020, the cumulative rate of infection among the population of The Gambia ≈1.5/1,000 persons (Figure 3, panel A). During the same period, 115 COVID-19 deaths were recorded across the country.

Among MRCG staff, stratified analysis showed that infection rates among CSD staff were 2.6 times higher than among non-CSD staff, whom we considered representative of the infection risk among the general population (Figure 3, panel B). By the end of September, the cumulative risk for infection among non-CSD MRCG staff was ≈124/1,000 persons (Figure 3, panel B). All 191 confirmed cases among MRCG staff were either asymptomatic or mildly symptomatic; no cases met WHO criteria for moderate or severe pneumonia and no deaths occurred in this cohort.

## Discussion

The COVID-19 pandemic arrived in The Gambia in July 2020, later than in most countries in the world. The Gambia had a short and intense first wave; 67% of cases occurred in August, and most cases were asymptomatic or mild. Among our MRCG cohort, 1/7 (14.3%) persons were SARS-CoV-2–positive. During the epidemic peak, the SARS-CoV-2 positivity rate among the population of The Gambia was >20%.

The later start of the epidemic is probably the result of the early closure of national borders, including for air travel, and of the identification and isolation of infected persons who continued to enter the country from Senegal. These measures were complemented by contact tracing and by the provision of facilities for quarantine by the government. The relative effects of these measures, together with other measures implemented during the state of emergency, such as closure of schools, reduction of access to markets, banning of large gatherings including at religious festivals, and use of facemasks, are hard to quantify, as are behavioral changes, such as social distancing and handwashing. Nonetheless, these measures seem to have been key in preparing the country to respond and minimize potential harm. 

The sudden increase of cases in August coincided with the major Muslim feast of Eid-Ul Adha, locally called Tobaski, on July 30, 2020, during which travel and family gatherings were common. However, the number of COVID-19 cases had already started to increase in July. 

Although climate in The Gambia is hot throughout the year, the peak epidemic coincided with the months of highest daily humidity and highest minimum temperature but lowest maximum temperature (*14*,*15*). Data on how temperature and humidity affect transmission are contradictory (*24*,*25*). In The Gambia, climate conditions might have had an indirect effect on transmission because persons are more likely to spend time indoors during the rainy season. In The Gambia, the rainy season also occurs during the months with the highest respiratory virus transmission (*26*).

Through the systematic testing of the MRCG staff cohort, including asymptomatic contacts and mildly symptomatic cases, we might have more robust estimates of the actual rates of SARS-CoV-2 infection in The Gambia than are available from the general population. The rate of SARS-CoV-2 in MRCG staff outside the CSD (124 cases/1,000 persons) was >80-fold higher than that reported for the general population. Rates among MRCG staff remained >40-fold higher than the general population, even when we considered only the more intensely populated coastal area of The Gambia in the denominator. Assuming the urban adult population had similar exposures and transmission as our MRCG cohort, we would expect >75,000 infections among the 601,394 persons 20–64 years of age who live in main towns. This estimation contrasts sharply with the 3,579 cases reported during the same period across the country and in all age groups, a discrepancy that could be partly explained by the high occurrence of asymptomatic or mildly symptomatic infections and the national testing strategy that used passive case detection and targeted symptomatic persons. Because >50% of the population is <20 years of age, we would expect a high frequency of asymptomatic infections in The Gambia. Indeed, the discrepancies between estimated and reported cases we noted are consistent with recent seroprevalence studies from eastern and southern Africa. Those studies suggest higher rates of community infection compared with those estimated by passive case surveillance. For instance, 3 weeks after the COVID-19 peak in South Africa, 40% of HIV-positive pregnant women had SARS-CoV-2 antibodies (*12*). In Kenya, a retrospective survey of blood donor samples collected during April–June 2020 found that 1 in 20 adults had SARS-CoV-2 antibodies (*13*). In Malawi, SARS-CoV-2 seroprevalence was 12.3% in a cohort of 500 healthcare workers sampled during May–June 2020; using the observed seroprevalence, the researchers concluded that the predicted number of deaths was 8 times the number of reported deaths (*11*). In a smaller study of 113 frontline healthcare workers in Nigeria, 45% had SARS-CoV-2 antibodies (*27*). In The Gambia, >30% of the CSD staff became infected by September 30, 2020. Rates among CSD staff were higher than the rest of the MRCG cohort, which probably reflects a combination of stronger surveillance and occupational clinical exposure exacerbated by traveling to work, but the weight of each factor is difficult to estimate. However, higher seroprevalence has been reported among healthcare workers in Europe (*28*).

The prevalence of mild disease also is reflected by the low occupancy of hospital beds reserved for severe COVID-19 patients. However, the fewer hospitalizations also could indicate avoidance of SARS-CoV-2 testing because of stigmatization, which has been observed in other regions (*7*). Indeed, among the 115 COVID-19 deaths counted in The Gambia, 30% of SARS-CoV-2 tests were performed postmortem on samples collected from patients hospitalized in non–COVID-19 health facilities. Without an official registration system for deaths, the overall toll of COVID-19–associated deaths is difficult to quantify, and the real number could be several times higher.

The low occurrence of severe disease in Africa compared with other continents underlines the importance of minimizing the potential collateral damage of the COVID-19 pandemic. Such damage includes diversion of financial and personnel resources from other services to the COVID-19 response, changes in healthcare seeking behavior, reduced availability of medicines for acute and chronic diseases, and disruption of routine vaccination services (*29*–*33*). The pandemic also has worsened the economic stability of households and increased food insecurity, particularly in low- and middle-income countries (*34*), and mitigating the short- and mid-term effects of the pandemic should be a priority. Use of COVID-19 restriction measures to control transmission must be carefully weighed against the economic effects these measures have on the population. Tackling fear and stigma will be essential to avoid decreases in health system use in future COVID-19 waves.

One limitation of our study is that, although cases in the general population and the MRCG cohort showed similar timelines and the size of the MRCG cohort is relatively large, MRCG cases could be considered a cluster. In addition, the level of education and the monthly income of MRCG staff is above that of the general population, thus, staff likely understand and are able to better implement prevention measures. MRCG staff live mainly in urban areas, where transmission tends to be higher (*35*), but they also live in less crowded environments with better access to water and sanitation, which could protect them from infection. MRCG also developed policies, launched many levels of staff education on COVID-19, and reinforced messages related to social distancing, handwashing, and the wearing of face masks at work and in the community. Given the nature of the MRCG’s work, the level of understanding and background knowledge of infectious diseases, even among staff not directly involved in research, likely is higher than for the general population. The rapid identification and isolation of cases through the robust surveillance among MRCG staff should have further limited transmission. On the other hand, no moderate or severe COVID-19 cases occurred among the MRCG staff. The mild clinical manifestations among cases were not modified by treatment; for instance, no MRCG staff member met WHO criteria for hospitalization and fewer required oxygen supplementation or dexamethasone treatment. The prevalence of risk factors for severity should be similar between MRCG staff and the population, except the MCRG cohort had fewer persons >60 years of age, which is a primary risk factor for severe COVID-19 and death. 

In conclusion, SARS-CoV-2 transmission in The Gambia was intense over a short period. Reassuringly, COVID-19 seems less severe in The Gambia than in high-income countries in Europe, North America, and Asia. It is unclear whether a second wave of infection will occur because the causes of the sudden increase of cases in July are unclear. We strongly encourage continuous protection of healthcare workers with appropriate PPE and strengthening of surveillance systems around the country to promptly detect another sudden increase of cases. Countrywide seroprevalence surveys would clarify the epidemiology of infection in different age groups and places. However, engaging with the community to mitigate collateral damage of the pandemic should take priority. In addition, investigation is needed to define the major drivers that shape the epidemic so differently in Africa than in some high-income regions. Clarifying such drivers should help model adequate interventions for both low- and high-income countries.
